# Supplementary material for: A fog-assisted group-based truth discovery framework over mobile crowdsensing data streams
Source: PLoS One. 2025 Aug 26;20(8):e0330656. doi: 10.1371/journal.pone.0330656 (PMC12380339; doi:10.1371/journal.pone.0330656)
Supplement: Attachment — Submitted filename: Response to Reviewers.docx [file pone.0330656.s001.docx]

**Reply Letter**

**[Tributes]**

We would like to express our deep thanks and gratitude to the reviewers for their comments on the paper. We have tried our best to modify the paper according to their recommendations. We hope our paper will be published in PLOS ONE.

**COMMENTS and Answers**

Reviewer:1

**Comment: 1**.      In the first Introduction, innovation points are proposed to be combined to no more than 3.

### **Answer**: We appreciate the reviewer’s comment. We re-write the contributions in three points as follows, the “Introduction” section, Page 3.

### “We develop a novel TD method for crowdsensing data streams where we initialized the weights in TD with participants’ credibility level. Then we formulate the credibility function based on the participants’ readiness, commitment, and the device’s ability to perform the sensing tasks. This method guarantees the accuracy of the data as participants with a high credibility level are likely to provide accurate data.

### We develop a novel fog-assisted Two-layer Group-based Truth Discovery (TGTD) mechanism. The first layer estimates the truth of the group’s members and the second layer estimates the aggregated truth for the groups in the area of interest. Additionally, it offloads the process to the FN deployed in a different geographic location, to minimize the overhead on the participants’ side.

### We conduct extensive simulations using synthetic datasets and different real-world datasets and show that the proposed framework outperforms the baseline TD approach.”

**Comment: 2**      Detailed analysis is suggested in the Design goals.

**Answer:** Thanks to the reviewer for the comment. Based on the comment, we added these paragraphs to the “Design Goals” Section, Page 8, and the “Performance Metrics” Section, Page 15.

“In this article, we intend to devise a TGTD mechanism, which can provide more accurate ground truth for group sensing activities. Specifically, our mechanism achieves the following twofold design goals.

1. Accuracy: The proposed scheme should output a highly accurate estimation. Accordingly, it is measured by the deviation between the estimated results and the real truths.

2. Efficiency: The proposed scheme should reach a significantly lower overhead for the participants. Thus, it is measured by the running time of the system to show the scalability and efficiency”

“1. Accuracy

To evaluate the deviation between the estimated results and the real truths. Hence, we measure the resulting accuracy by adopting the standard root of mean squared error (RMSE) [50] of estimated truth against the ground truth, according to:

The lower the value of RMSE, the better the performance of the scheme. Therefore, to measure if our approach can obtain a more accurate estimation result compared to other approaches. Hence, the smaller the value between the estimated results and the real truths, the higher the score the approaches get.

2. Efficiency

Measured by the running time of the system to show the scalability and efficiency of the framework, the lower the better. Through this matrix, we can see the computing overhead of our approach, which can prove the practicality of the approach.”

**Comment: 3:** In each figure containing subgraphs, (()) should be changed to ().

**Answer:** Thanks to the reviewer for this comment. We recheck the figures and change the format accordingly for all figures, from pages 16 to 19.

**Comment: 4:** The discussion or comparisons with more recent related schemes, such as ppru: a privacy-preserving reputation updating scheme for cloud-assisted vehicular networks, a privacy-preserving and reputation-based truth discovery framework in mobile crowdsensing, a lightweight privacy preservation scheme with efficient reputation management for mobile crowdsensing in vehicular networks, instead of conventional schemes are suggested.

**Answer:** Thanks to the reviewer for this comment. We added this paragraph to explain why we compare with the baseline iCRH algorithm. To the Section “Performance Evaluation”, Pages 13, and 14.

“**Performance Evaluation**

In this section, the TGTD algorithm over the data stream is evaluated by conducting various experiments and simulations on both real-world datasets and Synthetic datasets. We conduct a comparison between our scheme and the baseline framework for incremental truth discovery on streaming data iCRH [16], Algorithm (2). The iCRH proves its state-of-the-art efficiency performance in our target real-time MCS scenario. Similar to iCRH, our approach scans readings once for each time slot and has fewer computation steps. However, compared to iCRH which is for individual participant scenarios, our approach is for group MCS scenarios”.

**Note:** All the truth discovery frameworks are either based on the CRH algorithm for one-time sensing tasks or iCRH for stream sensing tasks. Hence, as we target stream MCS tasks we compare our algorithm with iCRH.

**Comment: 5:** The paper lacks theoretical analysis, and it is suggested to supplement.

**Answer:** We appreciate the comment for theoretical analysis, we added this paragraph at the end of the “Proposed Framework” Section, Page 13.

“The running time for the TGTD algorithm is O(G) for the while loop to go through all the groups (line 5). Then the for-loop to to go through all the group members (line 6), takes O(i). Therefore, TGTD is bounded by O(G ∗ i). Hence, the TGTD algorithm is computationally efficient.”

**Note:** We prove the efficiency and effectiveness of the framework through experiments on both Synthetic and two real-world datasets.

Reviewer:2

**Comment: 1:** What is the basis for selecting the proposed method compared to other methods? Suggested additional explanation.

**Answer:** Thanks to the reviewer for this comment. We added this paragraph to the “Performance Evaluation” Section on page 14.

“The iCRH proves its state-of-the-art efficiency performance in our target real-time MCS scenario. Similar to iCRH, our approach scans readings once for each time slot and has fewer computation steps. However, compared to iCRH which is for individual participant scenarios, our approach is for group MCS scenarios.”

**Comment: 2:** There is still room for improvement in English writing.

**Answer:** We appreciate the reviewer’s comment regarding improvement in English writing. We checked and corrected minor typos and long sentences in the revised manuscript.

Reviewer:3

**Comment: 1:** In the Introduction section, the “We proposed fog-assisted TD for crowdsensing systems” should be corrected to “We propose fog-assisted TD for crowdsensing systems”.

**Answer:** Thanks to the reviewer for this comment. We corrected the typo in the “Introduction” Section accordingly on page 3.

**Comment:** 2**:** Some explanation should be added to the Design Goals section.

**Answer:** We appreciate the reviewer’s comment. We added these paragraphs to the “Design Goals” Section, Page 8, and the “Performance Metrics” Section, on page 15.

“In this article, we intend to devise a TGTD mechanism, which can provide more accurate ground truth for group sensing activities. Specifically, our mechanism achieves the following twofold design goals.

1. Accuracy: The proposed scheme should output a highly accurate estimation. Accordingly, it is measured by the deviation between the estimated results and the real truths.

2. Efficiency: The proposed scheme should reach a significantly lower overhead for the participants. Thus, it is measured by the running time of the system to show the scalability and efficiency”

“1. Accuracy

To evaluate the deviation between the estimated results and the real truths. Hence, we measure the resulting accuracy by adopting the standard root of mean squared error (RMSE) [50] of estimated truth against the ground truth, according to:

The lower the value of RMSE, the better the performance of the scheme. Therefore, to measure if our approach can obtain a more accurate estimation result compared to other approaches. Hence, the smaller the value between the estimated results and the real truths, the higher the score the approaches get.

2. Efficiency

Measured by the running time of the system to show the scalability and efficiency of the framework, the lower the better. Through this matrix, we can see the computing overhead of our approach, which can prove the practicality of the approach.”

**Comment:** 3**:** The truth discovery process usually exposes the privacy of entities. Can this scheme achieve privacy protection? It is suggested that the author add some discussion on this aspect.

**Answer:** We appreciate the reviewer’s comment regarding the privacy issues, but as we mentioned at the end of the “Related work” Section, Pages 6 and 7, the following statement.

“In addition, we adopt a non-private version of the iCRH in this work.”

Hence, the privacy concern is out of the scope of this paper.

**Comment: 4:** The discussion or comparisons with more recent related schemes, such as a privacy-preserving and reputation-based truth discovery framework in mobile crowdsensing, a lightweight privacy preservation scheme with efficient reputation management for mobile crowdsensing in vehicular networks, efficient anonymous authentication and privacy-preserving reliability evaluation for mobile crowdsensing in vehicular networks, lightweight and privacy-preserving dual incentives for mobile crowdsensing, instead of conventional schemes are suggested.

**Answer:** Thanks to the reviewer for this comment. We added this paragraph to explain why we compare with the baseline iCRH algorithm. To the Section “Performance Evaluation”, Pages 13, and 14.

“**Performance Evaluation**

In this section, the TGTD algorithm over the data stream is evaluated by conducting various experiments and simulations on both real-world datasets and Synthetic datasets. We conduct a comparison between our scheme and the baseline framework for incremental truth discovery on streaming data iCRH [16], Algorithm (2). The iCRH proves its state-of-the-art efficiency performance in our target real-time MCS scenario. Similar to iCRH, our approach scans readings once for each time slot and has fewer computation steps. However, compared to iCRH which is for individual participant scenarios, our approach is for group MCS scenarios”

**Note:** All the truth discovery frameworks are either based on the CRH algorithm for one-time sensing tasks or iCRH for the stream sensing task. Hence, as we target stream MCS tasks we compare our algorithm with iCRH.

**Comment: 5:** two-layer group-based truth discovery (TGTD) mechanism” should be corrected to “Two-layer group-based truth discovery (TGTD) mechanism”.

**Answer:** Thanks to the reviewer for this comment. We rechecked the whole paper and corrected it to the “Two-layer Group-based Truth Discovery (TGTD) mechanism” accordingly.
