# Supplementary material for: A fog-assisted group-based truth discovery framework over mobile crowdsensing data streams
Source: PLoS One. 2025 Aug 26;20(8):e0330656. doi: 10.1371/journal.pone.0330656 (PMC12380339; doi:10.1371/journal.pone.0330656)
Supplement: Attachment — Submitted filename: Response_to_Reviewers_auresp_2.docx [file pone.0330656.s002.docx]

**Reply Letter**

**[Tributes]**

We would like to express our deep thanks and gratitude to the reviewers for their comments on the paper. We have tried our best to modify the paper according to their recommendations. We hope our paper will be published in PLOS ONE.

**COMMENTS and Answers**

Reviewer:1

**Comment: 1**.      It is suggested that the author follow the reviewer's opinion and make careful revisions before submitting the paper for review again.

**Answer**: Thank you for your feedback. We apologize if our previous revisions did not fully address the reviewer’s comments. We have now carefully reviewed and revised the paper, ensuring that all suggestions and concerns have been thoroughly addressed. All the changes have been highlighted in Yellow. We appreciate your patience and look forward to your further feedback on our revised submission.

Reviewer:3

**Comment: 1**.      The authors should add a discussion or comparison with the latest options listed below to make your paper more convincing.

### **Answer**: We appreciate the reviewer’s comment. In response, we have re-experimented and compared our approach with the latest streaming truth discovery approach, Cen-CATD [1] in addition to the baseline approach [2]. Based on the reviewer’s feedback, we have revised the Evaluation section, which now spans pages 16 to 21.

### **Note:** The list of papers mentioned in the comments does not address dynamic data truth discovery algorithms (i.e., they do not consider timestamps of the data, but rather focus on convergence criteria). Both iCRH and our algorithm, TGTD, iterate over each timestamp of streaming/dynamic data, rather than using a loop with convergence-stopping criteria. Therefore, comparing algorithms that rely on timestamps with those that use convergence criteria is not fair. To ensure a fair comparison, we have identified the latest streaming truth discovery algorithms and re-experimented them, comparing our algorithm with two other streaming truth discovery approaches.

[1]	P. S. Mukkamala, H. Wu, and B. Düdder, "Reliable and Streaming Truth Discovery in Blockchain-based Crowdsourcing," in *2023 20th Annual IEEE International Conference on Sensing, Communication, and Networking (SECON)*, 2023, pp. 492-500: IEEE.

[2]	Y. Li *et al.*, "Conflicts to harmony: A framework for resolving conflicts in heterogeneous data by truth discovery," *IEEE Transactions on Knowledge and Data Engineering,* vol. 28, no. 8, pp. 1986-1999, 2016.
