# Supplementary material for: A fog-assisted group-based truth discovery framework over mobile crowdsensing data streams
Source: PLoS One. 2025 Aug 26;20(8):e0330656. doi: 10.1371/journal.pone.0330656 (PMC12380339; doi:10.1371/journal.pone.0330656)
Supplement: Attachment — Submitted filename: Response_to_Reviewers_auresp_4.docx [file pone.0330656.s003.docx]

**Reply Letter**

**[Tributes]**

We would like to express our deep thanks and gratitude to the reviewers for their comments on the paper. We have tried our best to modify the paper according to their recommendations. We hope our paper will be published in PLOS ONE.

**COMMENTS and Answers**

Reviewer#4

**Comment: 1**.      While the proposed work is valuable and I would recommend for publication, but, the authors should clearly state in the abstract and in the Proposed Framework section that fog architecture simulation, streaming process organisation are not their primary contributions and have not been fully analysed, and these areas of research are areas for further investigation.

**Answer**: We thank the reviewer for the valuable comment. In response, we have revised the abstract to include the following statement: **'The organization of the streaming process within the fog architecture simulation is identified as an area for further investigation and future work**.'

Moreover, in the Proposed Framework section, we stated that ‘**However, the analysis of fog architecture simulation and the organization of streaming processes were beyond the scope of this study, as they do not constitute its primary contributions. These topics are identified as valuable directions for future research and in-depth investigation**.’

Additionally, we have updated the conclusion by expanding the future work section to state: **'In future work, we aim to address the organization of the streaming process in the context of fog architecture simulation**.'

Reviewer#5

**Comment: 1**.    The manuscript has been revised, and it is more suitable for publication to the best of my knowledge.

**Answer**: Thank you very much, we sincerely appreciate that.
